# Feasibility and diagnostic accuracy of Telephone Administration of an adapted wound heaLing QuestiONnaire for assessment for surgical site infection following abdominal surgery in low and middle-income countries (TALON): protocol for a study within a trial (SWAT)

**DOI:** 10.1186/s13063-021-05398-z

**Published:** 2021-07-21

**Authors:** NIHR Global Health Research Unit on Global Surgery, James Glasbey, Victoria Adeyeye, Adesoji Ademuyiwa, Alisha Bhatt, Bruce Biccard, Jane Blazeby, Peter Brocklehurst, Sohini Chakrabortee, Jean De La Croix Allen Ingabire, Francis Moïse Dossou, Irani Durán, Rohini Dutta, Dhruv Ghosh, Frank Gyamfi, Parvez Haque, Pollyanna Hardy, Gabriella Hyman, Ritu Jain, Oluwaseun Ladipo-Ajayi, Ismail Lawani, Souliath Lawani, Mwayi Kachapila, Karolin Kroese, Rachel Lillywhite, Rhiannon Macefield, Laura Magill, Janet Martin, Jonathan Mathers, Punam Mistry, Rohin Mittal, Mark Monahan, Rachel Moore, Dion Morton, Faustin Ntirenganya, Emmanuel Ofori, Rupert Pearse, Alberto Peón, Thomas Pinkney, Antonio Ramos de la Medina, Tubasiime Ronald, David Roman, Anita Slade, Stephen Tabiri, Donna Smith, Aneel Bhangu

**Affiliations:** NIHR Global Health Research Unit on Global Surgery, University of Birmingham, Institute of Translational Medicine, Heritage Building, Mindelsohn Way, Birmingham, B15 2TH UK

**Keywords:** Digital follow-up, Surgical site infection, Telephone follow-up, Outcome assessment, Trial retention, Trial methodology, Patient-reported outcome measure, Abdominal surgery, Global surgery, Surgery

## Abstract

**Background:**

Surgical site infection is the most common complication of abdominal surgery, with a global impact on patients and health systems. There are no tools to identify wound infection that are validated for use in the global setting. The overall aim of the study described in this protocol is to evaluate the feasibility and validity of a remote, digital pathway for wound assessment after hospital discharge for patients in low- and middle-income countries (LMICs).

**Methods:**

A multi-centre, international, mixed-methods study within a trial, conducted in two stages (TALON-1 and TALON-2). TALON-1 will adapt and translate a universal reporter outcome measurement tool (Bluebelle Wound Healing Questionnaire, WHQ) for use in global surgical research (SWAT store registration: 126) that can be delivered over the telephone. TALON-2 will evaluate a remote wound assessment pathway (including trial retention) and validate the diagnostic accuracy of this adapted WHQ through a prospective cohort study embedded within two global surgery trials. Embedded community engagement and involvement activities will be used to optimise delivery and ensure culturally attuned conduct. TALON-1 and TALON-2 are designed and will be reported in accordance with best practice guidelines for adaptation and validation of outcome measures, and diagnostic test accuracy studies.

**Discussion:**

Methods to identify surgical site infection after surgery for patients after hospital discharge have the potential to improve patient safety, trial retention, and research efficiency. TALON represents a large, pragmatic, international study co-designed and delivered with LMIC researchers and patients to address an important research gap in global surgery trial methodology.

**Supplementary Information:**

The online version contains supplementary material available at 10.1186/s13063-021-05398-z.

## Background

### Importance of surgical site infection research

Surgical site infection (SSI) is a global issue. It is the most common healthcare-associated infection in low- and middle-income countries (LMICs) [1, 2] and carries a huge burden to patients, doctors, and health systems around the world [3–5]. Reported rates vary, but SSI is particularly prevalent in abdominal and colorectal surgery; as many as one in three patients get an SSI when the operation involves the large bowel [6]. It was highlighted as the key research priority to improve surgical care worldwide in an international prioritisation process [7] and is the focus of several ongoing global randomised trials [8–11].

### Challenges to assessment of wound infection across settings

The current ‘gold standard’ for diagnosis of SSI in randomised trials is in-person review according to the Centre for Disease Control Criteria at 30 days after surgery by a trained assessor [2]. Hospitals in LMICs treat a high burden of surgical disease [12] and have a high number of eligible patients for recruitment to pragmatic clinical trials. However, in-person assessment is labour and time intensive and requires patients to take additional time-off work and incur costs of travel. This poses particular difficulty in LMICs where patients may live further from a specialist hospital and may already be at risk of financial catastrophe as a result of their index procedure [13, 14]. The SARS-CoV-2 pandemic poses an additional risk where patients are returning to hospital in the perioperative setting [15, 16]. Other methods for assessing SSI are important.

Over 80% of the global population has access to a mobile telephone, opening an opportunity for remote and digital wound assessment pathways [17, 18]. Non-standardised telephone follow-up may risk reducing the validity of outcome assessment. For example, whilst 43% of patients in a prospective cohort study underwent telephone-based assessment, this group had a significantly lower risk-adjusted odds ratio of SSI than those who underwent in-person follow-up [6]. Quality assured methods for remote wound evaluation are urgently required, both to deliver high-quality research and for surveillance after hospital discharge.

### Potential solutions for remote surgical site assessment

The Bluebelle Wound Healing Questionnaire (WHQ) has been developed and validated in the UK (English language) to assess post-discharge infections following abdominal surgery (HTA: 12/200/04) and is attractive for use in randomised trials. The WHQ was designed to be either completed by a healthcare professional or self-reported by patients [19] and as such has been described as a ‘universal-reporter’ outcome measure (UROM) [20]. In a UK validation study, the WHQ demonstrated good reliability and excellent discrimination [21–23]. The WHQ was completed both in-person and over the telephone by a healthcare professional trained in wound assessment (e.g. nurse, junior doctor), demonstrating feasibility of telephone delivery. However, no external validation has been performed in LMICs where health literacy, language and cultural contexts, and digital infrastructure differ substantially. If the WHQ can be administered remotely (e.g. over the telephone) with satisfactory diagnostic accuracy, this would reduce resource usage, making surgical research more effective and more sustainable. Other digital adjuncts to surgical site evaluation such as video assessment may further enhance accuracy [24].

### Justification of study design

Studies within a trial (SWATs) have gained significant attention from trial methodologists and funders over the past 3 years and are now the focus of a Trial Methodology Research Partnership working group (Trial Forge) [25] and National Institute for Health Research (NIHR) funding stream. SWATs exploit the delivery network and infrastructure of major randomised trials to efficiently answer methodological research questions. Ongoing large international trials in global surgery provide a unique opportunity to improve the quality and efficiency of global wound infection research [26].

The overall aim of this study protocol is to evaluate the feasibility and validity of a remote, digital pathway for wound assessment after hospital discharge for patients in low-resource settings.

## Methods/design

### Design summary

Feasibility and diagnostic accuracy of *T*elephone *A*dministration of an adapted wound hea*L*ing Questi*ON*naire for assessment for surgical site infection following abdominal surgery in low and middle-income countries (TALON) is a prospective, multi-centre, international, non-randomised study embedded within a randomised trial, conducted in two phases (TALON-1 and TALON-2). TALON-1 will adapt and translate an outcome measure for use in global surgical research. TALON-2 will validate this adapted outcome measure through a prospective cohort study embedded within two host trials in global surgery.

### Host trials

FALCON is a stratified, pragmatic, multi-centre, 2 × 2 factorial trial testing two measures (skin preparation and antimicrobial sutures) to reduce superficial or deep skin infection following abdominal surgery in seven low- and middle-income countries (NCT03700749) [8]. ChEETAh is a cluster randomised trial evaluating whether the practice of using separate sterile gloves and instruments to close wounds at the end of surgery compared to current routine hospital practice can reduce surgical site infection at 30 days after abdominal surgery [26].

### Reporting and registration

This protocol is reported with reference to recommendations from the Global Health Network for qualitative research in LMICs and Consolidated criteria for reporting qualitative research (COREQ) framework [27, 28], recommendations for best practices in a mixed-methods adaptation of outcome measures [29], and STARD guidelines for diagnostic test accuracy studies [30]. The protocols for the two host trials are reported elsewhere [8, 26]. This SWAT protocol has been pre-registered on the MRC Hubs for Trial Methodology Research Study Within a Trial database [25] (Queen’s University Belfast) (SWAT ID:126).

### Ethics and approvals

The TALON-1 and TALON-2 studies have been approved as an amendment to the host trial protocols by the University of Birmingham Research Ethics Committee (ERN_18-0230_A and ERN_19-0719). The additional risks and ethical implications within TALON-2 were considered very low by the Birmingham Clinical Trials Unit internal review board and international ethics committee. Ethical approval for TALON has been obtained from national, regional, and/or hospital-level ethics committees for selected centres in all participating countries, in accordance with local protocols.

## TALON-1

### Study objectives

1. To assess patient acceptability, cross-cultural and cross-language equivalence, and content validity of the Wound Healing Questionnaire (WHQ) across LMICs
2. To assess the scaling and psychometric properties of the WHQ when used across different patient populations and subgroups
3. To adapt the WHQ for use in global surgical research by triangulating qualitative and quantitative data

### Study design

TALON-1 will use mixed qualitative and quantitative research methods to explore the cross-cultural and cross-language equivalence of the WHQ across settings, and the acceptability of telephone-based follow-up. Iterative adaptation to the questionnaire will be made, where required, to create an adapted patient-reported outcome measure suitable for use in global trials. The original WHQ prior to adaptation is presented in Additional file 1. Scaling and measurement functioning of the WHQ will be evaluated within a pilot cohort study. Interview data will be triangulated with data about the psychometric properties of the WHQ using Rasch Unidimensional Measurement Modelling.

### Language and local acceptability

All TALON study researchers are fluent in the English language. In some countries, English is a primary or prevalent secondary language amongst the populations that will be recruited to FALCON. In these countries, the feasibility of single-language administration of the questionnaire has been tested at sites within the FALCON trial. Where translation of the WHQ is required, this will be performed using the Mapi process for standard linguistic validation to verify conceptual equivalence across languages and cultures [31–33].

In brief, this involves recruitment and briefing of an in-country consultant to oversee the process in the target country; forward translation by two independent translators native in both the target and source language; production of a reconciled language version with discussion between the translators where warranted; review of the forward translation by the consultant; backward translation into the source language by an independent translator fluent in the target and source language; comparison of the backward translation and original, analysis of discrepancies, and reconciliation with decisions reported and explained; review of the backward translation by the consultant; pilot testing; and finally clinician review.

As the WHQ will be translated into multiple languages, an international harmonisation meeting with the consultants overseeing each language translation will be held after all translations are completed in order to ensure conceptual equivalence in all versions.

### Questionnaire adaptation

Whilst cognitive debriefing with patients is the recommended methodology for cross-language adaptation of an outcome measure [33, 34], modification will be required to progress the study during the SARS-CoV-2 pandemic. As such, adaptation will start with expert review and structured interviews with site researchers. Structured interviews will be conducted with two to three research staff that are participating in the FALCON trial (research nurses, or doctors involved in follow-up) in each participating country in order to ensure cross-cultural relevance of concepts and construct validity of the questionnaire. First, unrefined data from each interview will be reviewed. Second, structured item-by-item summaries will be generated for each interview according to a pre-defined template from the Social Research Association. Third, themes related to comprehension, response mapping, retrieval, and judgement will be extracted with flexibility to include emerging findings. Finally, themes will be categorised for each item to compare and contrast these across interviews. Where required, iterative adaptation will be made until a point of saturation according to accepted best practice principles for adaptation of instruments [29, 33, 35].

Further iterative adaptation will be performed using cognitive interviews undertaken face-to-face with patients if this is safe and practicable. Otherwise, adaptation will use data from site researchers only, with this limitation noted in study outputs. Interview data will be used to explore content, conceptual and cross-cultural equivalence of the global WHQ in greater depth [35]. Data will be collected up to the point of saturation; we anticipate approximately 6 to 8 patients per country will be sufficient. Adult patients (> 18 years) that have undergone intra-abdominal surgery of any urgency and through any operative approach will be recruited from surgical assessment areas, inpatient wards, and outpatient clinic settings. Informed consent for inclusion will be taken and recorded within a dedicated Informed Consent Form. A specific Patient Information Sheet for TALON-1 will be provided. Purposive sampling will include patients from each included country, those who have and have not suffered an SSI, urban and rural hospital settings, and high school level of education and lower education level. The results of cognitive debriefing will be reviewed to assure cultural relevance and equivalence. Comparison of patients’ interpretation of the translation and the original version will be highlighted to amend discrepancies.

### Psychometric testing and scaling

Pilot administration of the WHQ will be performed in adult patients undergoing abdominal surgery and recruited to the host trials, over the telephone by a non-surgeon clinician; this can be a research nurse, non-surgeon physician, or other delegated members of the site research team. Non-surgeon physicians will be trained to deliver the WHQ by a member of the study management group. Monitoring of the first 10 patients followed up over that telephone at each site will be performed for quality assurance. Content, construct validity, and unidimensionality of the WHQ will be tested using Rasch analysis [36]. The Rasch unidimensional measurement model will examine the psychometric properties of the WHQ, identify anomalies in the data, and evaluate the extent to which the WHQ items are measuring wound infection [37, 38]. Differential item functioning will be tested for equivalence by country, language, age (young age (18–29)/middle age (30–59)/older age (> 60 years)), and sex (male/female) groups. A target of at least 100 patients per language and per country will be set for data included in the Rasch analysis [39].

Data sources will be triangulated using data (i.e. between countries) and methodological (i.e. between qualitative interviews and psychometric analysis of quantitative pilot data) triangulation to support the final adaptation of the WHQ across each included language [29, 40–43]. This will be followed by proofreading, before completion of a final report and publication of the adapted WHQ.

### Community engagement and involvement

Community engagement and involvement with patients and members of the public from LMICs will be engaged in all phases of the design and delivery of TALON-1. The interview topic guide will be co-designed with input from a representative global surgery patient forum. Practicable methods for conducting interviews, and patient compensation for the time in participation will be determined with the support of local community leaders. The Guidance for Reporting Involvement of Patients and the Public (GRIPP-2) short form will be used to track and report the impact of community engagement and involvement (CEI) within this study [44].

### Ethical considerations and data handling

All participant data for TALON-1 will be fully anonymised and unlinked and stored securely within a password-protected NVivo V12 data management system.

TALON-1 will therefore produce a globally adapted Wound Healing Questionnaire, suitable for use in global surgery research, including the TALON-2 validation study.

## TALON-2

TALON-2 will be a cohort study within the FALCON and ChEETAh trials to test the feasibility and accuracy of telephone administration of the adapted WHQ in the diagnosis of surgical site infection.

### Study objectives

1. To evaluate the diagnostic accuracy of telephone administration of the WHQ in detection of abdominal surgical site infection across LMICs
2. To assess the feasibility of delivery of the telephone WHQ by a non-surgeon researcher within the FALCON trial
3. To assess the feasibility of live wound videography (or wound photography) as a diagnostic adjunct for telephone-based wound follow-up

### Centre selection

**Fig. 1:**
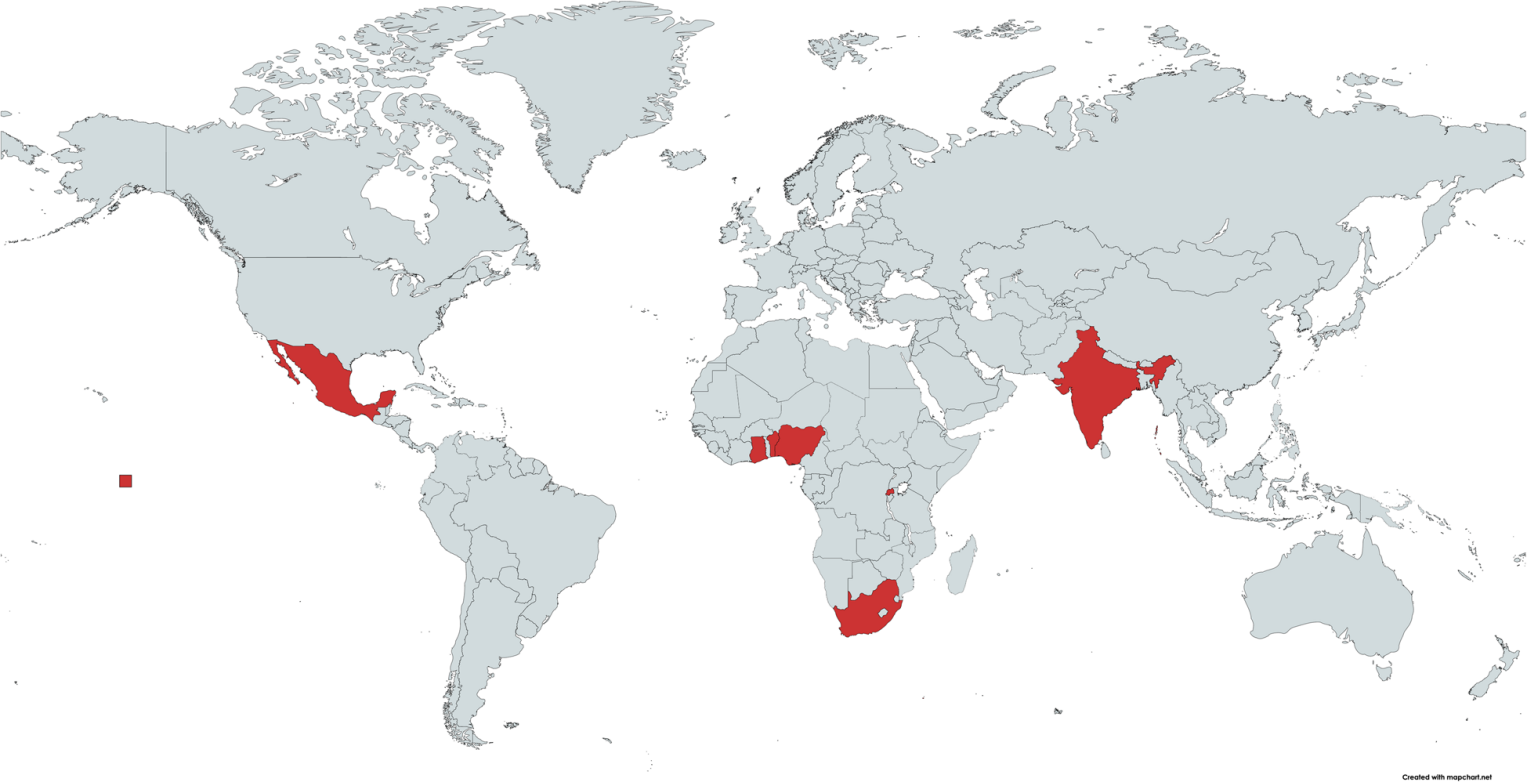
Map of participating countries. Participating countries marked in red

Centres for TALON-2 will be chosen based upon (1) the proportion of recruited patients that are likely to return for routine in-person 30-day follow-up and (2) the site’s ability to have an independent non-surgeon researcher perform telephone follow-up for TALON-2 that will not perform in-person, 30-day follow-up. Sites will be selected from across seven low- and middle-income countries (Fig. 1).

### Eligibility criteria

Consecutive adult patients (greater than 18 years) recruited to the FALCON or ChEETAh study that are likely to return for routine in-person follow-up at around 30 days postoperatively are eligible. Consent for an additional telephone follow-up call within the TALON-2 study will be taken at the same time as trial consent, using a targeted Informed Consent Form and Patient Information Sheet. Community representatives will co-produce these resources to ensure culturally appropriate language and delivery.

### Study intervention

**Fig. 2:**
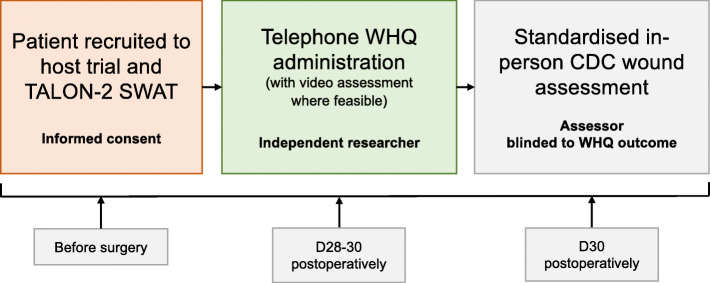
TALON-2 study with a trial patient pathway. WHQ=Bluebelle Wound Healing Questionnaire, SWAT=study within a trial, D30 postoperative day 30 with day 0 as date of surgery

Telephone-based administration of the WHQ will be compared to the ‘gold standard’ (reference test) of in-person assessment at 30 days after surgery by a trained clinician in accordance with the US Centre for Disease Control criteria (Additional file 2). The WHQ will be delivered integrated into the trial pathway for included patients (Fig. 2). The telephone-based WHQ will be performed at 28–30 days (i.e. in the 72 h prior to in-person follow-up) by a non-surgeon researcher, according to a telephone script. Patients will be asked to provide between one and three telephone contact numbers, either personal or belonging to a family member or community worker. The non-surgeon researcher directing completion of the WHQ should be blinded to the outcome of the in-person wound assessment within the FALCON trial. In the event that the patient is unable to be contacted by telephone at 27–30 postoperative days (before in-person follow-up), the WHQ can be performed after the in-person follow-up appointment, where possible. This should be completed by a non-surgeon researcher that is independent of the assessment for the FALCON primary outcome, to ensure independent measures are taken. The process and pathway for telephone follow-up will be co-designed by patient and public partners to optimise successful and culturally sensitive delivery.

After sufficient data has been collected for the primary validation analysis, an extension to the study intervention will be implemented. Patients will undergo remote telemedical (photo or video) review to track their recovery from surgery and will be invited by the assessor to provide ‘live’ visualisation of the wound to check wound healing (Additional file 3). This will be performed using a locally available video platform as an adjunct to completion of the WHQ in remote wound assessment. No recording or storage of the video data will be undertaken.

### Telephone follow-up pathway

Descriptive data to explore variation in telephone follow-up implementation and procedures between patient groups, centres, and countries will be collected including the number of attempts required to connect, the type of phone used (including whether the phone has a camera or video capability), the phone’s owner, and the language of delivery.

### Primary outcome measure

- Proportion of surgical site infections that are correctly identified by the telephone WHQ (in comparison to in-person review), summarised using measures of diagnostic test accuracy

### Secondary outcome measures

The secondary outcome measures will test the feasibility of telephone and video follow-up and specifically the benefit to trial retention of remote methods (i.e. maintenance of a patient with trial follow-up to the end of the study, in this case 30 days after surgery).

- Retention benefit: ratio of the proportion of recruited patients returning a telephone WHQ to the proportion of recruited patients completing in-person follow-up
- Telephone contact rate: proportion of patients successfully contacted by the telephone
- Return rate: the proportion of telephone WHQ returned, and reasons not completed
- Data completion rate: proportion of missing data within each form
- Video contact rate: proportion of patients successfully contacted for a live video wound assessment

### Sample size

As TALON-2 is a validation study for a diagnostic criterion, no formal sample size calculation is required. A range of sample sizes and their impact on the 95% confidence interval (CI) around estimates of sensitivity 0.92 and specificity 0.95 based on a prevalence of 0.21 using the binomial exact formula are presented in Table 1. Sample sizes are adjusted to allow for 15% loss to follow-up from the host trials, and 15% of patients who do not achieve both telephone WHQ completion and 30-day in-person follow-up within TALON-2. Sampling from Hub and Spoke centres across several LMICs will give a representative sample of urban and rural settings.

**Table 1:** TALON-2 study sample size estimates

Patients recruited to host trial	Patients retained for follow-up	Paired WHQ and in-person follow-up	Patients with SSI^a^	Patients without SSI^b^	Precision around test accuracy measures	
					Sensitivity (95% C.I.)	Specificity (95% C.I.)
714	607	516	108	408	0.92 (0.85–0.96)	0.95 (0.93–0.97)
571	485	412	87	325	0.92 (0.84–0.97)	0.95 (0.92–0.97)
428	364	309	65	244	0.92 (0.83–0.97)	0.95 (0.92–0.97)
285	242	206	43	163	0.92 (0.81–0.99)	0.95 (0.91–0.98)

^a^0.21 * number paired WHQ and in-person follow-up

^b^0.79 * number paired WHQ and in-person follow-up

### Community engagement and involvement

We will work with patient and public partners to co-produce the question schedule, introductory and closing text, and patient facing materials. Using recommendations from focus group discussions with community representative and local research leaders, we will create a toolkit to optimise telephone follow-up in low-resource settings which can be applied across the TALON delivery network.

### Statistical analysis

Statistical analysis will be undertaken using R Project for Statistical Computing (V3.6.1). The outcome against which the WHQ prediction model will be validated is 30-day in-person wound assessment according to the CDC criteria (binary outcome: SSI/no SSI). Blinded 30-day outcome data for patients included in TALON will be made available by the FALCON Trial Management Group for the purpose of this analysis. Cross-tabulations of the reference CDC diagnosis (‘no SSI’ or ‘SSI’) and a binary variable of the self-assessment WHQ total score (created by a cut-off score; for instance, a WHQ total score of less than or equal to a specified value) will be compared.

Criterion validity will be examined against the reference (face-to-face SSI assessment) to evaluate the performance of WHQ in discriminating between individuals with and those without SSI. Sensitivity and 1-specificity values of the WHQ for different cut-off scores will be used to plot a receiver operating characteristic (ROC) curve. From derivation data in the UK, WHQ cut points of 6 to 8 were found to provide optimal sensitivity and specificity [21]. We will calibrate the tool with a range of cut points from 5 to 9 in order to explore differences in calibration across included countries. The overall ability of the WHQ to discriminate between individuals with and those without SSI will be measured by the area under the ROC curve (AUC), with uncertainty presented using 95% confidence intervals. Diagnostic test accuracy will be presented as sensitivity, specificity, and positive and negative predictive value.

The overall rate of missing data is anticipated to be low. The patients that did and did not receive a 30-day in-person assessment (reference standard) will be compared to assess for partial or differential verification bias. A sensitivity analysis will be performed with missing data imputed using multiple imputation by chained equations to explore the impact of missing data. A full statistical analysis plan has been published online at: https://globalsurg.org/resources/phd-research-projects/talon/.

### Ethics and governance

For TALON-2, all patient identifiable data (including telephone numbers) will be held at host trial sites on an encrypted, password-protected spreadsheet and only used for the purpose of telephone follow-up within the host trial and TALON studies. Data for TALON-2 will be collected on the existing, secure REDCap system created for the host trials.

Live, real-time ‘video’ wound assessment within the TALON-2 study will be conducted in countries/environments where this is already used as part of routine clinical follow-up pathways, learning from experiences during the COVID-19 pandemic. This will use a locally available video platform, and the costs of data usage will be borne by the clinical research team and not by the participant themselves. No recording of video data will be made or stored. Video wound assessment will be entirely voluntary at the discretion of the participant, and the wound assessor will encourage the patient to perform the wound assessment in a private and secure environment at home or in the community. Only the wound assessor (and trained clinical translator where required) will be present for the video assessment and the patient will be encouraged; this mirrors current clinical practice and standards for an in-person wound assessment. A standard operating procedure and online training materials will be provided as an adjunct to site initiation training to standardise as much of this assessment as possible. The patient will be free to terminate the video at their own discretion at any time.

### Dissemination

All publications arising from TALON will be attributed to the NIHR Global Health Research Unit on Global Surgery [6, 45, 46]. All contributors will be listed as collaborating authors in accordance with the National Research Collaborative recommendations [47]. Data sharing will be made available upon successful completion of a Data Sharing Agreement and approval from the TALON Study Management Group.

Lay summaries of all research outputs will be co-produced with CEI partners and translated into relevant languages before dissemination. Other scientific engagement methods such as online webinars, visual and video abstracts, national journal editorial submissions, and policy reports will be implemented with LMIC partners across participating countries.

## Discussion

TALON is a large international collaborative cohort study which will evaluate a telephone follow-up pathway for surgical site assessment following abdominal surgery across seven low- and middle-income countries. This will allow researchers to better understand both the feasibility and validity of remote follow-up for SSI across diverse settings. Remote follow-up methods are an attractive target for improving trial retention; this study is designed to compare the within-participant attrition rates between in-person and telephone follow-up in-depth. Through adaptation and translation of an existing patient-reported outcome measure, TALON will provide a package of tools for surgical researchers across cultural and language contexts. Finally, TALON will adopt a novel and efficient study within a trial (SWAT) design, which provides an attractive future model for methodological research in global surgery.

Methods of post-discharge follow-up which do not require face-to-face review have grown rapidly in interest over the past 5 years [48, 49]. Exponential advances in global access to telecommunications and mobile devices have been bolstered by a global momentum towards telemedical delivery of postoperative care during the SARS-COV-2 pandemic [50–52]. The World Bank estimates that over 80% of the population of sub-Saharan Africa now have access to a mobile telephone, and this continues to increase year-on-year [18]. Remote follow-up has several advantages to patients in LMICs and research studies. Firstly, patients can avoid significant additional costs of transport and time out of work associated with return to hospital [53]. Secondly, telemedicine may reach patients who are unable to return to the hospital for reasons of costs, access, or logistics, improving trial retention [6, 24, 54, 55]. Thirdly, it can protect scarce time for busy LMIC clinicians and improve trial efficiency [56]. However, rapid implementation of unvalidated digital follow-up methods into randomised trials (or indeed clinical practice) may risk harm to patients through missed infection, overtreatment, and/or introducing research bias [24]. Existing evidence is available only from small, low-quality studies at high risk of bias and/or without adopting a diagnostic accuracy frame of evaluation [54, 57–60]. This large, pragmatic study aims to fill this knowledge gap by exploring the feasibility and accuracy of a novel remote follow-up pathway.

SSI is the most common complication of surgery, and the third most common healthcare-associated infection worldwide [1, 2]. The global burden of morbidity as a result of surgical infection has dramatic and wide-reaching effects on patients, providers, and health systems in LMICs [14, 61, 62]. SSI has therefore been recognised as the highest global priority in surgical research and is therefore a natural target for the TALON study [7, 63]. Future work will be required to standard processes for outcome assessment across other common and severe postoperative complications [64]. TALON provides a proof-of-concept for international SWATs which can now be used to explore other prioritised methodological challenges in global surgery trials.

The TALON study has been designed as a deep collaboration between global surgery clinicians, researchers, methodologists, and community and patient partners. This focus will be essential to assuring appropriate, culturally attuned, and therefore successful delivery of the study [65, 66]. Understanding of community engagement and involvement in global health research is still evolving [67]; learning from this study will hope to inform future research from our group and provide case studies to support other global health researchers.

There are limitations to the proposed study design. Firstly, due to limitations with travel around the SARS-CoV-2 pandemic, the ability to conduct cognitive interviewing during the adaptation and translation process may be limited. From site researchers and CEI representatives, it was highlighted that video-based interviews with patients in low-resource settings may prove challenging both logistically, and to the richness of the data, and risk of accentuating power imbalances between the researcher and patients. We have modified the proposed methodology to include pragmatic, structured interviews with site researchers; however, further iterative adaptation following face-to-face patient interviews may be required in the future [29]. A mixed-methods approach, including Rasch analysis of pilot cohort data, will further enhance the adaptation and scaling process [68]. Secondly, the Wound Healing Questionnaire has not previously been validated for use across low-resource settings, as such the optimal score calibration is yet to be determined. Whilst a pre-defined range of cut points will be adopted, this combines the calibration and validation phases into a single analysis; this was a pragmatic decision to manage a complex project across global trials in a manageable timeframe. Thirdly, we have not pre-defined thresholds for sensitivity and specificity for that would be acceptable for clinical practice or trial adoption in this protocol. Consensus work is required to define minimally acceptable criteria for diagnostic accuracy during future development and implementation of the WHQ [69]. Fourthly, the WHQ can be administered any time during the 72 h preceding in-person assessment. Whilst there is a theoretical risk that patients could suffer a new SSI event 28 to 30 days after surgery that has not previously manifested, this is clinically improbable and is not supported by existing data [6, 70]. Another risk is that completion of telephone follow-up immediately prior to in-person follow-up may have a deleterious effect on in-person follow-up rates, overestimating the retention benefit. We have designed the TALON case report form and patient information to stress the need for both in-person and telephone follow-up, and can compare rates of attrition to the overall host trial population. Finally, the exploratory use of video follow-up in this study is not the central focus of this work. Whilst this will provide the first available data on video-based follow-up of surgical wounds in LMICs, contributing significantly to understanding of this area, further work is likely to be required to standardise protocols for ‘live’ video assessment and explore its reliability in depth.

## Study status

At the time of submission (12 February 2021), adaptation and translation of the WHQ in TALON-1 has been completed. The FALCON trial has completed recruitment, and data from 1400 patients for the pilot cohort study in TALON-1 has been obtained for use in the Rasch analysis. Data for the TALON-2 validation study has been obtained for 350 patients to date; recruitment to the ChEETAh trial and TALON-2 validation study is ongoing.

## Supplementary Information

**Additional file 1.** Index test: Original Wound Healing Questionnaire first developed by Macefield et al. (J Infect Prev, 2017).

**Additional file 2.** Reference standard: diagnostic criteria for surgical site infection used in FALCON and ChEETAh trials.

**Additional file 3.** Extension for live video assessment of surgical wound.

## Data Availability

Upon completion of the TALON-1 and TALON-2 studies, data will be made available to other investigators upon request. This will be dependent upon the completion of a Data Sharing Agreement.

## Abbreviations

SWAT: Study within a trial
WHQ: Wound Healing Questionnaire
UROM: Universal reporter outcome measure
TALON: Feasibility and diagnostic accuracy of Telephone Administration of an adapted patient-reported wound heaLing QuestiONnaire for assessment of surgical site infection following abdominal surgery in low and middle-income countries
FALCON: Pragmatic multi-centre factorial randomised controlled trial testing measures to reduce surgical site infection in low- and middle-income countries
ChEETAH: Cluster randomised trial of sterile glove and instrument change at the time of wound closure to reduce surgical site infection. A trial in low- and middle-income countries
SSI: Surgical site (wound) infection
