# Supplementary material for: Feasibility and diagnostic accuracy of Telephone Administration of an adapted wound heaLing QuestiONnaire for assessment for surgical site infection following abdominal surgery in low and middle-income countries (TALON): protocol for a study within a trial (SWAT)
Source: Trials. 2021 Jul 21;22:471. doi: 10.1186/s13063-021-05398-z (PMC8293583; doi:10.1186/s13063-021-05398-z)
Supplement: Supplementary file 1 — Additional file 1. Index test: Original Wound Healing Questionnaire first developed by Macefield et al. (J Infect Prev, 2017). [file 13063_2021_5398_MOESM1_ESM.docx]

**Additional file 1. Index test: Original Wound Healing Questionnaire first developed by Macefield et al. (J Infect Prev, 2017).**

**FALCON Substudy (TALON-2): Wound Healing Questionnaire**

**For questions with tick boxes, please tick one box per question.**

The Wound Healing Questionnaire should be completed between 27-30 days after the patient’s operation over the telephone, before they undergo standard 30-day wound assessment as part of the FALCON trial. **The Wound Healing Questionnaire should not be completed by the same person that will complete the standard 30-day FALCON Follow-up Form.**

|  | | | FALCON Trial Number | | | | | | |  | | | |  | | | | |  | | |  | | |  | | | | |  | | | |
| --- | --- | --- | --- | --- | --- | --- | --- | --- | --- | --- | --- | --- | --- | --- | --- | --- | --- | --- | --- | --- | --- | --- | --- | --- | --- | --- | --- | --- | --- | --- | --- | --- | --- |
|  |  |  | Centre name | | | | | | | ____________________________ | | | | | | | | | | | | | | | | | | | | | | | |
|  |  |  | Patient name | | | | | | | ____________________________ | | | | | | | | | | | | | | | | | | | | | | | |
| **Patient status** | | | | | | | | | | | | | | | | | | | | | | | | | | | | | | | | | |
| Has the patient died? | | | | - Yes (***please stop at Patient Status***) | | | | | | | | | | | | | | | - No (continue to Follow-up details) | | | | | | | | | | | | |  |  |
| If patient died, date of death | | | | d | | d | m | m | y | | | y | y | | | y | | | ***If patient died, an SAE form must be completed*** | | | | | | | | | | | | |  |  |
| If patient died, main cause of death | | | | _________________________________________________ | | | | | | | | | | | | | | | | | | | | | | | | | | | |  |  |
| **Follow-up details** | | | | | | | | | | | | | | | | | | | | | | | | | | | | | | | |  |  |
| Were you able to contact the patient by telephone? | | | | - Yes (please continue) | | | | | | | | | | | | | | | - No (***please stop here***) | | | | | | | | | | | | |  |  |
| If telephone contact was made, date of contact | | | | d | | d | m | m | y | | | y | y | | | y | | |  | | | | | | | | | | | | | | |
| *Ask the patient:*  What type of phone are they using for this call? | | | | - Landline phone | | | | | | | | | | | | | - Mobile phone (without a camera) | | | | | | | | | | | | | | | | |
|  |  |  |  | - Mobile phone (with a camera) | | | | | | | | | | | | |  |  |  |  |  |  |  |  |  |  |  |  |  |  |  |  |  |
| *Ask the patient:*  Who owns the phone I have called you on? | | | | - Patient themselves | | | | | | | | | | | | | - Friend or relative | | | | | | | | | | | | | | | | |
|  |  |  |  | - Healthcare worker | | | | | | | | | | | | | - Other (please specify):   _______________________ | | | | | | | | | | | | | | | | |
| *Ask the patient:*  Do you live in an urban (city or town) or rural (countryside) area? | | | | - Urban | | | | | | | | | | | | | - Rural | | | | | | | | | | | | | | | | |
| *Ask the patient:*  What is your highest level of education? | | | | - High school or above | | | | | | | | | | | | | - Below high school level | | | | | | | | | | | | | | | | |
| What language did the patient use to respond to the questionnaire? | | | | - English | | | | | | | | | | | | | - Other (please specify):   _______________________ | | | | | | | | | | | | | | | | |
| *If other*: Was the formal translated Questionnaire used? | | | | - Yes, formal questionnaire | | | | | | | | | | | | | - No, translated by questionnaire administrator | | | | | | | | | | | | | | | | |
| **Please read the following statement to the patient:**  We are interested in knowing how your wound(s) have healed since you left hospital after your surgery. It is fine to ask someone else to help answer some of the questions, for example if you cannot easily see your wound(s). If you have more than one wound, please answer the questions thinking about just one wound — especially if there have been any concerns about how it has been healing. We would like you to think about the wounds on your skin. Some of the questions I am about to ask you relate to some problems that may occur with wound healing. Please note, many people do not experience these problems after having surgery.  **Since you left hospital after having surgery…** | | | | | | | | | | | | | | | | | | | | | | | | | | | | | | | | | |
| Was there redness spreading away from the wound? | | | | - Not at all | | | | | - A little | | | | | | | | | | - Quite a bit | | | | | | | - A lot | | | | | | | |
| Was the area around the wound warmer than the surrounding skin? | | | | - Not at all | | | | | - A little | | | | | | | | | | - Quite a bit | | | | | | | - A lot | | | | | | | |
| Has any part of the wound leaked clear fluid? | | | | - Not at all | | | | | - A little | | | | | | | | | | - Quite a bit | | | | | | | - A lot | | | | | | | |
| Has any part of the wound leaked blood-stained fluid? | | | | - Not at all | | | | | - A little | | | | | | | | | | - Quite a bit | | | | | | | - A lot | | | | | | | |
| Has any part of the wound leaked thick and yellow or green fluid? | | | | - Not at all | | | | | - A little | | | | | | | | | | - Quite a bit | | | | | | | - A lot | | | | | | | |
| Have the edges of any part of the wound separated or gaped open of their accord? | | | | - Not at all | | | | | - A little | | | | | | | | | | - Quite a bit | | | | | | | - A lot | | | | | | | |
| If the wound edges opened, did the deeper tissue also separate? | | | | - Not at all | | | | | - A little | | | | | | | | | | - Quite a bit | | | | | | | - A lot | | | | | | | |
| Has the area around the wound become swollen? | | | | - Not at all | | | | | - A little | | | | | | | | | | - Quite a bit | | | | | | | - A lot | | | | | | | |
| Has the wound been smelly? | | | | - Not at all | | | | | - A little | | | | | | | | | | - Quite a bit | | | | | | | - A lot | | | | | | | |
| Has the wound been painful to touch? | | | | - Not at all | | | | | - A little | | | | | | | | | | - Quite a bit | | | | | | | - A lot | | | | | | | |
| Have you had, or felt like you have had, a raised temperature or fever (>38^o^C)? | | | | - Not at all | | | | | - A little | | | | | | | | | | - Quite a bit | | | | | | | - A lot | | | | | | | |
| Have you sought advice because of a problem with your wound, other than at a planned follow-up appointment? | | | | | | | | | | | | | | | | | | | | - Yes | | | | | | | - No | | | | | |  |
| Has anything been put on the skin to cover the wound? (dressing) | | | | | | | | | | | | | | | | | | | | - Yes | | | | | | | - No | | | | | |  |
| Have you been back into hospital for a problem with your wound? | | | | | | | | | | | | | | | | | | | | - Yes | | | | | | | - No | | | | | |  |
| Have you been given antibiotics for a problem with your wound? | | | | | | | | | | | | | | | | | | | | - Yes | | | | | | | - No | | | | | |  |
| Have the edges of your wound been deliberately separated by a doctor or nurse? | | | | | | | | | | | | | | | | | | | | - Yes | | | | | | | - No | | | | | |  |
| Has your wound been scraped or cut to remove any unwanted tissue? | | | | | | | | | | | | | | | | | | | | - Yes | | | | | | | - No | | | | | |  |
| Has your wound been drained? (drainage of pus or an abscess) | | | | | | | | | | | | | | | | | | | | - Yes | | | | | | | - No | | | | | |  |
| Have you had an operation under general anaesthetic for treatment of a problem with your wound? | | | | | | | | | | | | | | | | | | | | - Yes | | | | | | | - No | | | | | |  |
| **Before you end the call,** inform the patient that **this telephone questionnaire will not replace the 30-day in-person wound assessment** required as part of the FALCON trial, and **they must still attend their in-person follow-up appointment (or receive an additional telephone call** if this is not possible). | | | | | | | | | | | | | | | | | | | | | | | | | | | | | | | | |  |
| **Form completed by** | | | | | | | | | | | | | | | | | | | | | | | | | | | | | | | | | |
| Job role | - Surgeon | - Other doctor | | | - Nurse | | | | | | - Other (please specify): _____________ | | | | | | | | | | | | | | | | | | | | | |  |
| Print full name |  | | | | | | | | | | | | | | | | | | | | | | | | | | | | | | | | |
| Signature |  | | | Date form completed | | | | | | | | | | | d | | | d | | | m | | m | y | | | | y | y | | y | | |
