# Supplementary material for: Feasibility and diagnostic accuracy of Telephone Administration of an adapted wound heaLing QuestiONnaire for assessment for surgical site infection following abdominal surgery in low and middle-income countries (TALON): protocol for a study within a trial (SWAT)
Source: Trials. 2021 Jul 21;22:471. doi: 10.1186/s13063-021-05398-z (PMC8293583; doi:10.1186/s13063-021-05398-z)
Supplement: Supplementary file 2 — Additional file 2. Reference standard: diagnostic criteria for surgical site infection used in FALCON and ChEETAh trials. [file 13063_2021_5398_MOESM2_ESM.docx]

**Appendix B: Reference standard: diagnostic criteria for surgical site infection used in FALCON and ChEETAh trials**

The following US Centre for Disease Control Criteria definition will be used in the FALCON and ChEETAh to identify deep incisional or superficial incisional SSIs.

- The infection must occur within 30-days of the index operation

**AND**

- The infection must involve the skin, subcutaneous, muscular or fascial layers of the incision

**AND**

- The patient must have at least one of the following:
- Purulent drainage from the wound
- Organisms are detected from a wound swab
- Wound opened spontaneously or by a clinician AND, at the surgical wound, the patient has at least one of: pain or tenderness; localised swelling; redness; heat; systemic fever (>38°C).
- Diagnosis of SSI by a clinician or on imaging
