# Supplementary material for: Feasibility and diagnostic accuracy of Telephone Administration of an adapted wound heaLing QuestiONnaire for assessment for surgical site infection following abdominal surgery in low and middle-income countries (TALON): protocol for a study within a trial (SWAT)
Source: Trials. 2021 Jul 21;22:471. doi: 10.1186/s13063-021-05398-z (PMC8293583; doi:10.1186/s13063-021-05398-z)
Supplement: Supplementary file 3 — Additional file 3. Extension for live video assessment of surgical wound. [file 13063_2021_5398_MOESM3_ESM.docx]

**Appendix C. Extension for live video assessment of surgical wound**

| **TALON Video Wound Assessment** | | | |
| --- | --- | --- | --- |
| **At this point in the follow-up up telephone call you should perform the video assessment of the** **wound** (*if possible*).  This should involve:   - Confirmation of verbal consent - If the wound is current dressed, ensure the patient has a spare dressing to apply to the wound before starting. Ask the patient to remove any wound dressing or covering that is present. - A ‘live’ video examination of the wound along its length - Closer examination of the wound in any areas where there is a concern about healing - Asking the patient to gently manipulate the wound if required, to express any pus or demonstrate separating of wound edges - Use of the Centre for Disease Control criteria to identify whether a wound infection has occurred - No photo or video data should be recorded, stored or sent to the assessor as part of this assessment   The objective of the video wound assessment is to mimic in-person assessment as closely as possible. | | | |
| Was verbal consent for video wound assessment confirmed? | - Yes | - No (***go to TALON Wound Healing Questionnaire***) | |
| Were you able to successfully complete a video assessment? | - Yes | - No | |
| *If no:* Why were you unable to complete the assessment?  (*Tick all that apply*) | - Poor signal | - Patient unable to use camera | |
|  | - Low quality image | - Patient unhappy to continue assessment | |
|  | - Other (please specify): ________________ | | |
| Was there evidence that the patient has got or has had a wound infection from the video assessment of the wound? | - Yes | | - No |
| Was there an ongoing wound infection at the time of assessment? | - Yes | | - No |
